# Early Production of the Neutrophil-Derived Lipid Mediators LTB_4_ and LXA_4_ Is Modulated by Intracellular Infection with* Leishmania major*

**DOI:** 10.1155/2017/2014583

**Published:** 2017-10-18

**Authors:** Michael Plagge, Tamás Laskay

**Affiliations:** Department of Infectious Diseases and Microbiology, University of Lübeck, Lübeck, Germany

## Abstract

Recruitment of neutrophil granulocytes to sites of infectious tissue damage is an early event in innate immune responses. Following chemotactic signals neutrophils establish a first line of defense in a swarm-like manner. Intracellular pathogens such as* Leishmania major* can, however, evade neutrophil-mediated killing and survive inside neutrophils. To achieve this the parasites evolved potent evasion mechanisms. Since neutrophils are a major source of inflammation regulating lipid mediators, we hypothesized that intracellular infection modifies the release of pro- and anti-inflammatory lipid mediators like leukotriene B4 (LTB_4_) and lipoxin A4 (LXA_4_), respectively. In the present study, we demonstrated* in vitro* that* L. major*-infected primary human neutrophils release an increased amount of LTB_4_, whereas LXA_4_ liberation is reduced during the first hours of infection. To investigate whether lipid mediator modulation is a common feature in intracellular infections, we tested the impact of an infection with* Anaplasma phagocytophilum*. Similarly to* L. major*, neutrophil infection with* A. phagocytophilum* led to an enhanced release of LTB_4_ and decreased LXA_4_ production. Together, our findings indicate that intracellular infections modulate the lipid mediator profile of neutrophils. This effect is likely to contribute to the survival of the pathogens in neutrophils and to the outcome of the infections.

## 1. Introduction

As the most numerous cell type in early acute inflammation, polymorphonuclear neutrophil granulocytes (PMN) can be central choreographers of inflammation [1–4]. During this phase, they occupy an outstanding position in the regulation of local lipid mediators, a family of mainly arachidonic-acid-derived signal molecules with potent effects on leukocyte recruitment and activity [5, 6]. The proinflammatory lipid mediator leukotriene B4 (LTB_4_) works as an amplifier for localized inflammatory signals and was shown to be critical for sufficient PMN recruitment, termed swarming,* in vivo* [7]. In addition, LTB_4_ was shown to enhance phagocytic activity, activation, degranulation, and killing of internalized pathogens [8–11]. Since PMN are the predominant source of LTB_4_, they promote acute inflammation in a feed-forward manner [12, 13]. In addition to their proinflammatory functions neutrophils also contribute to the resolution of inflammation. To inhibit overwhelming inflammation, the LTB_4_-precursor LTA_4_ is used for the synthesis of proresolving lipid mediators (SPMs). This process, termed class switch, is characterized by the production of lipoxin A_4_ (LXA_4_), a prototype member of SPMs [5, 14]. LXA_4_ is known to inhibit leukocyte chemotaxis, transmigration, ROS-generation, NF-kB activation, and synthesis of proinflammatory cytokines [15, 16]. LXA_4_ is mainly produced by transcellular dual lipoxygenation. In this process PMN-derived LTA_4_ is used as a substrate of 12- and 15-LO expressed in epithelial cells [17, 18]. Therefore, neutrophils not only produce the proinflammatory lipid mediator LTB_4_ but also contribute to the production of the anti-inflammatory mediator LXA_4_. Upon exposure to the calcium ionophore ionomycin the LTB_4_ and LXA_4_ production by neutrophils can be regarded as a measurement of the cells' capacity for the synthesis of these lipid mediators. By using LPS + fMLP information regarding the LTB_4_ release by neutrophils in an infected/inflammatory environment can be obtained.

Certain pathogenic microorganisms such as the protozoan parasite* Leishmania major* (*L. major*) can evade destruction and survive inside neutrophils [19].* L. major* is an obligatory intracellular parasite and causative agent of Old World cutaneous Leishmaniasis. During blood meal the parasites are transmitted into the skin of mammalian hosts by the bite of infected sandflies. Neutrophils are rapidly recruited to the site of* Leishmania* infection and phagocytose the parasites [20, 21]. In this context,* L. major* parasites were shown to exploit the early inflammatory response by using PMN as transient host cells [22–25]. Lipid mediators have been shown to be involved in the survival strategy of* Leishmania* parasites [26–29]. However, no data concerning the impact of* L. major* infection on neutrophil-derived lipid mediators is available.

In the present study, we investigated how intracellular infection with* L. major* affects the release of the pro- and anti-inflammatory lipid mediators LTB_4_ and LXA_4_ by primary human neutrophil granulocytes* in vitro*. In addition to* L. major*, we tested the impact of an infection with* Anaplasma phagocytophilum* (*A. phagocytophilum)* on LTB_4_- and LXA_4_-synthesis to investigate whether the neutrophil lipidome is a common target of intracellular pathogens.* A. phagocytophilum* is an obligate intracellular bacterium and causative agent of the tick-borne Human Granulocytic Anaplasmosis (HGA). It is critically dependent on neutrophils as its definitive host cells and well known for host cell modulations that lead to subversion of PMN antimicrobial defense mechanisms to ensure intracellular survival [30].

## 2. Materials and Methods 

### 2.1. Ethics

Blood collection was conducted with the understanding and written consent of each participant and was approved by the ethical committee of the Medical Faculty of the University of Lübeck (05-124).

### 2.2. Isolation of Human Peripheral Blood Neutrophil Granulocytes

Peripheral blood was collected in lithium-heparin-containing tubes. Neutrophils were isolated in a combination of two density gradient centrifugations as described previously [31]. Using layered lymphocyte separation medium 1077 (PAA, Pasching, Austria) and Histopaque 1119 (Sigma-Aldrich, Deisenhofen, Germany) first, the separated granulocytes were purified in a discontinuous Percoll (Amersham Biosciences, Uppsala, Sweden) gradient centrifugation. After isolation neutrophils were resuspended in complete medium (RPMI-1640 medium supplemented with 10% heat-inactivated fetal calf serum) and 50 *μ*M *β*-mercaptoethanol (all from Sigma-Aldrich, Steinheim, Germany), 4 mM L-glutamine, and 10 mM HEPES (both Biochrom, Berlin, Germany). The obtained cell preparations contained >99% granulocytes according to morphological examination of cytocentrifuge slides stained with Diff Quik (Medion Diagnostics, Düdingen, Switzerland).

### 2.3. Leishmania Major Culture

The origin and propagation of the cloned virulent* L. major* strain MHOM/IL/81/FEBNI has been described elsewhere [32]. In short,* L. major* promastigotes were cultured on biphasic rabbit blood agar-containing microtiter plates at 26°C in humidified atmosphere containing 5% CO_2_ for 7 to 10 days and a maximum of five passages. Each plate well contained 100 *μ*l liquid medium and 50 *μ*l of a Novy-MacNeal-Nicolle (NNN) blood agar slant, which was prepared by supplementing 200 ml of Brain-Heart-Infusion agar base (Difco, Detroit, MI, USA) with 50 ml defibrinated fresh rabbit blood (Elocin-Lab, Oberhausen, Germany). The liquid medium consisted of RPMI-1640 medium supplemented with 5% heat-inactivated fetal calf serum and 25 *μ*M *β*-mercaptoethanol (all from Sigma-Aldrich), 4 mM L-glutamine, 10 mM HEPES, 100 U/ml penicillin, and 100 *μ*g/ml streptomycin (all from Biochrom). For infection* L. major* promastigotes were washed with complete medium for 10 minutes at 2600 ×g. After centrifugation the supernatant was discarded and the pellet was resuspended in complete medium. Promastigotes with active flagellar movement were counted in a hemocytometer with a chamber depth of 0.02 mm.

### 2.4. Preparation of Cell-Free* Anaplasma phagocytophilum*

The* A. phagocytophilum* Webster strain was a kind gift of Dr. J. S. Dumler, John Hopkins University, Baltimore, MD. The bacteria were propagated and cell-free* A. phagocytophilum* was prepared as described previously [33]. Briefly, infected HL-60 cells were centrifuged at 250 ×g for 10 min and resuspended in 2 ml of PBS. Subsequently, cells were passed through a 25 G needle followed by a 27 G needle for 10 times each and vortexed with sterile solid glass-beads for 1 minute. By centrifuging at 750 ×g for 10 min cellular debris was removed. The supernatant was collected and centrifuged at 2.500 ×g for 15 min. The obtained cell-free* Anaplasma *containing pellets were used to infect neutrophils.

### 2.5. HT-29 Cell Culture

The human adenocarcinoma epithelial-like cell line HT-29 was kept in DMEM medium supplemented with 10% heat-inactivated FCS (both Sigma-Aldrich), 2 mML-glutamine, 100 U/ml penicillin, and 100 *μ*g/ml streptomycin (all from Biochrom) under humidified conditions at 37°C and 5% CO_2_. Placed in a well of a 96-well flat bottom microplate 9 × 10^4^ cells formed a confluent monolayer within 24 hours. Preliminary experiments showed that HT-29 cells do not release LTB_4_ nor LXA_4_ on their own (data not shown).

### 2.6. *In Vitro* Coincubation of Neutrophils with Pathogens and Determination of Infection Rate

Neutrophils (5 × 10^6^ per ml) were coincubated with* L. major* promastigotes or cell-free* A. phagocytophilum* in complete medium for 300 min at 37°C in humidified atmosphere containing 5% CO_2_. The multiplicity of infection (MOI) for* L. major* was 5. For infection with* A. phagocytophilum* the infectious load was 1 : 1 meaning that one neutrophil was infected with* A. phagocytophilum* obtained from one infected HL-60 cell. Subsequently, neutrophils were washed three times (400 ×g, 10 min) with PBS to remove noningested pathogens. Immediately before induction of lipid mediator release, the cells were resuspended in FCS-free complete medium since preliminary studies indicated a high FCS induced background signal in the LTB_4_-ELISA assay. The infection rates for* L. major* and* A. phagocytophilum* were determined by morphological examination of >200 PMN after Diff Quik staining of cytocentrifuge slides.* A. phagocytophilum* bacteria were visualized in neutrophils by immunocytochemical staining with the use of a polyclonal anti-*A. phagocytophilum *antibody (a kind gift of Professor J. Stephen Dumler, John Hopkins University, Baltimore, MD).

### 2.7. Induction and Measurement of LTB_4_

Prior to the induction of LTB_4_ release, the cells were resuspended in FCS-free complete medium. Infected and noninfected PMN as well as pathogen controls (*L. major* promastigotes or cell-free* A. phagocytophilum* without neutrophils) were exposed to either the combination of LPS and fMLP (1 *μ*g/ml LPS for 30 min followed by 0.5 *μ*M fMLP 10 min; both Sigma-Aldrich) or ionomycin (0.2 *μ*M, 10 min, Sigma-Aldrich). Control samples were left untreated. The induction was carried out in a 37°C water bath and terminated by cold centrifugation (4°C, precooled, 800 ×g, 10 min). The supernatants were collected and stored at −80°C. LTB_4_ was finally quantified by competitive enzyme-linked immunosorbent assay (R&D Systems, Minneapolis, MN, USA) according to the manufacturer's instructions.

### 2.8. Induction and Measurement of LXA_4_

Neutrophils were coincubated with* L. major* or with* A. phagocytophilum* for 300 min as described above. For the induction of LXA_4_ 10 ng/ml GM-CSF (Peprotech, Hamburg, Germany) was added as priming agent [34] for the last 90 minutes of incubation. The same procedure was applied to the pathogen controls. All cells were then washed and resuspended in FCS-free complete medium. The cells were then transferred to a 96-well flat bottom microplate with wells containing a confluent monolayer of HT-29 epithelial cells. The ratio of HT-29 cells to PMN in the coculture was approximately 1 to 7.5. For the induction of LXA_4_, infected and noninfected PMN as well as pathogen controls were treated with 1 *μ*M ionomycin (10 min or 360 min; Sigma-Aldrich) or left untreated. Stimulation was carried out at 37°C in humidified atmosphere containing 5% CO_2_ and terminated by centrifugation at 4°C (precooled, 800 ×g, 10 min). Supernatants were collected and stored at −80°C. LXA_4_ was finally quantified by ELISA (United States Biological, Salem, MA, USA) according to the manufacturer's instructions.

### 2.9. Statistical Analysis

Student's* t*-test for paired samples was performed in GraphPad Prism 7 software (La Jolla, CA, USA) for the comparison of lipid mediator content in the supernatants of infected and noninfected PMN after induction with ionomycin or LPS + fMLP. Data are presented as mean (±SD). Differences with a *p* value ≤ 0.05 were considered significant.

## 3. Results

### 3.1. Infection with* Leishmania major* Leads to Enhanced Production of LTB_4_ by Primary Human Neutrophils

**Figure 1:**
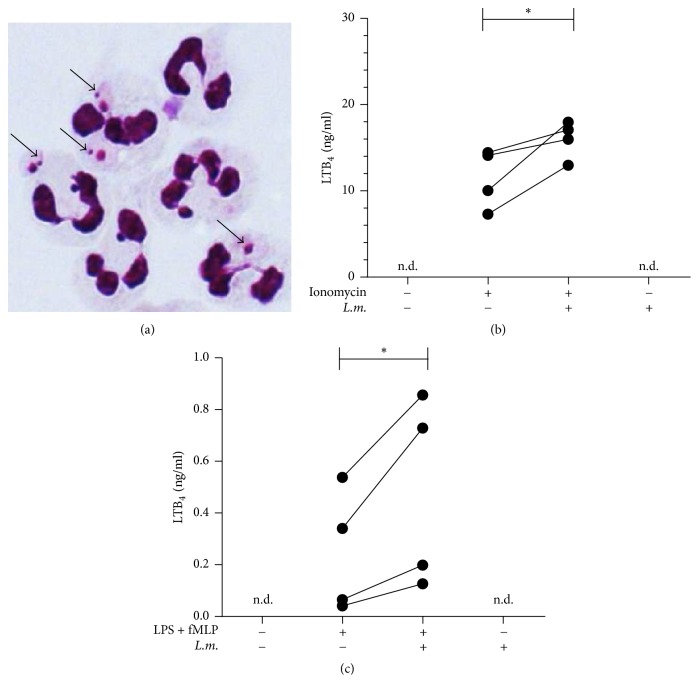
Infection with* L. major* leads to enhanced production of LTB_4_ by neutrophils. PMN were coincubated with* L. major* stationary phase promastigotes for 300 min at 37°C in humidified atmosphere containing 5% CO_2_ in complete medium. The multiplicity of infection (MOI) for* L. major* was 5. (a) The micrograph shows Diff Quik-stained neutrophils with internalized* L. major* promastigotes (arrows). (b)* L. major*-infected and uninfected neutrophils were treated with ionomycin (0.2 *μ*M, 10 min). LTB_4_ content of the supernatants was measured by ELISA. (c)* L. major*-infected and uninfected neutrophils were exposed to LPS (1 *μ*g/ml, 30 min) followed by fMLP (0.5 *μ*M, 10 min). LTB_4_ content of the supernatants was measured by ELISA. *n* = 4, n.d.: not detectable, and ^*∗*^*p* ≤ 0.05.

Primary human neutrophils were infected* in vitro* with stationary phase* L. major* promastigotes. After five hours of coincubation with the parasites the ratio of infected neutrophils was 65%  ±  15% as determined by examination of Diff Quik-stained slides (Figure 1(a)). Treatment with ionomycin or the combination of LPS and fMLP resulted in the release of LTB_4_ (Figures 1(b) and 1(c)). Infection with* L. major* significantly (*p* = 0.048) enhanced the ionomycin-induced LTB_4_ release from 11.5 (±3.4) ng/ml to 16.0 (±2.1) ng/ml (Figure 1(b)). After infection with* L. major* the LTB_4_-release induced by LPS + fMLP was also significantly (*p* = 0.049) enhanced from 0.24 (±0.23) ng/ml to 0.48 (±0.37) ng/ml (Figure 1(c)). The infection with* L. major* itself, without additional stimulation, did not result in the release of LTB_4_ (Figures 1(b) and 1(c)).

### 3.2. Infection with* Leishmania major* Results in Decreased Release of LXA_4_ by Neutrophils

**Figure 2:**
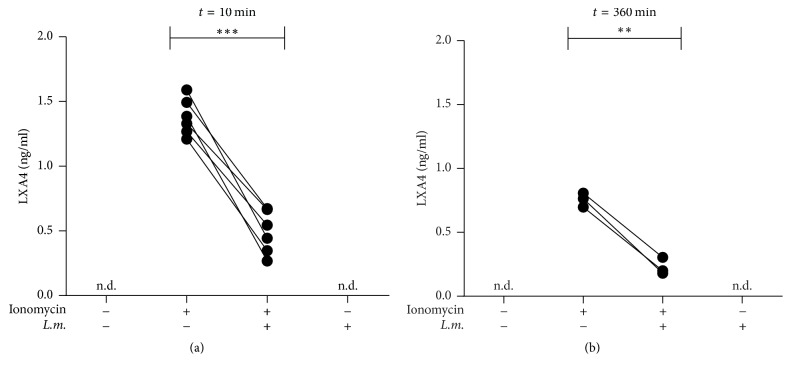
The release of LXA_4_ is reduced in* Leishmania major*-infected PMN. PMN were infected with* L. major* promastigotes for 300 minutes. During the last 90 min of infection 10 ng/ml GM-CSF was added.* L. major*-infected and uninfected neutrophils were coincubated with HT-29 cells and stimulated with ionomycin (1.0 *μ*M) for 10 (a) or 360 (b) min. LXA_4_ content of the supernatants was determined by ELISA. (a) *n* = 6, (b) *n* = 3, n.d.: not detectable, ^*∗∗*^*p* ≤ 0.01, and ^*∗∗∗*^*p* ≤ 0.001.

Neutrophil-mediated LXA_4_ production was assessed in a coculture assay with HT-29 epithelial cells. In this assay ionomycin induced a rapid production of LXA_4_ 10 minutes after induction with ionomycin (Figure 2(a)). At this point in time, infection with* L. major* led to a significant (*p* = 0,0001) reduction of LXA_4_ production from 1.38 (±0.14) ng/ml to 0.49 (±0.17) ng/ml (Figure 2(a)). Also 360 minutes after induction with ionomycin the production of LXA_4_ was significantly (*p* = 0.0027) reduced from 0.76 (±0.05) ng/ml in noninfected PMN to 0.23 (±0.07) ng/ml in* L. major*-infected neutrophils (Figure 2(b)). The infection with* L. major*, without additional stimulation, did not result in the release of LXA_4_ at both points in time (Figures 2(a) and 2(b)).

### 3.3. *Anaplasma phagocytophilum*-Infected Primary Human Neutrophils Show Increased LTB_4_ and Decreased LXA_4_ Release

**Figure 3:**
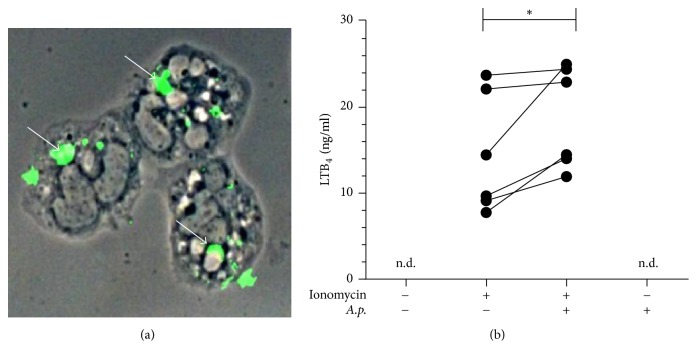
Infection with* Anaplasma phagocytophilum* leads to an enhanced release of LTB_4_. Primary human neutrophil granulocytes were coincubated with cell-free* A. phagocytophilum* for 300 min and extracellular bacteria were removed by washing. (a) Immunocytochemical staining of coincubated PMN reveals the presence of intracellular bacteria (arrows). (b)* A. phagocytophilum*-infected and noninfected neutrophils were treated with ionomycin (0.2 *μ*M, 10 min). LTB_4_ content of the supernatants was measured by ELISA. *n* = 6, n.d.: not detectable, and ^*∗*^*p* ≤ 0.05.

**Figure 4:**
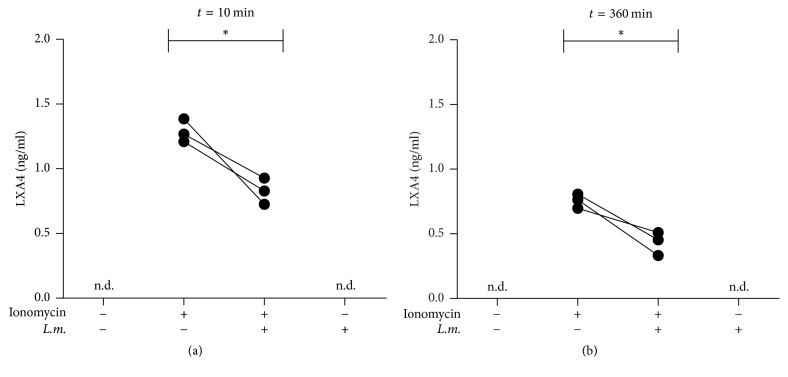
The release of LXA_4_ is reduced in* Anaplasma phagocytophilum*-infected PMN. Primary human neutrophil granulocytes were coincubated with cell-free* A. phagocytophilum* for 300 min and extracellular bacteria were removed by washing. During the last 90 min of infection 10 ng/ml GM-CSF was added. For transcellular lipoxin synthesis* A. phagocytophilum*-infected and uninfected neutrophils were coincubated with HT-29 cells and stimulated with ionomycin (1.0 *μ*M) for 10 (a) or 360 (b) min. LXA_4_ content of the supernatants was determined by ELISA. *n* = 3 each, n.d.: not detectable, and ^*∗*^*p* ≤ 0.05.

After coincubation with cell-free* A. phagocytophilum* for five hours, more than 95% of primary human neutrophils contained* A. phagocytophilum *bacteria (Figure 3(a)). Infection with* A. phagocytophilum* led to a significantly (*p* = 0.03) enhanced release of LTB_4_ by neutrophils 10 minutes after induction with ionomycin from 14.5 (±6.9) ng/ml to 18.8 (±5.9) ng/ml (Figure 3(b)). The release of LXA_4_ was significantly (*p* = 0.04) reduced in* A. phagocytophilum*-infected neutrophils at the same time point from 1.3 (±0.09) ng/ml to 0.8 (±0.1) ng/ml (Figure 4(a)). Six hours after induction with ionomycin a low LXA_4_ production was observed which was significantly (*p* = 0.04) inhibited by infection with *A*. phagocytophilum from a level of 0.76 (±0.05) ng/ml to 0.43 (±0.09) ng/ml (Figure 4(b)). The infection with* A. phagocytophilum* alone did not induce neither LTB_4_ nor LXA_4_ (Figures 3(b); 4(a), 4(b)).

## 4. Discussion

Lipid mediators are essential regulators of neutrophil granulocyte recruitment and activity [13, 35]. In this study we investigated the influence of intracellular infections on the early release of LTB_4_ and LXA_4_ by primary human neutrophils* in vitro*. We showed that infection with either* L. major* or* A. phagocytophilum *leads to an increased release of proinflammatory LTB_4_ whereas proresolving LXA_4_ is significantly reduced. Taken together, our data revealed a proinflammatory shift in PMN-derived lipid mediators during the early phase of intracellular infection with both pathogens. Limitations of this work are, however, the use of the epithelial tumor cell line HT-29 instead of primary cells, the limited numbers of experimental replications, and the* in vitro* approach itself.

With regard to the neutrophil-dependent establishment of productive* L. major* infections and the Trojan horse hypothesis [22, 23] our findings suggest that* L. major* parasites abuse infected PMN for augmented recruitment of neutrophils. By increasing neutrophil-derived LTB_4_ as a key recruitment factor [7] along with an upregulated IL-8 [36],* L. major* can ensure the sufficient presence of transient host cells for the subsequent infection of macrophages [22]. In accordance with this view, we could show a downregulation of the recruitment antagonist LXA_4_ during the first six hours of infection. As* L. major* parasites benefit from high doses of LXA_4_ in the later phase of inflammation [37], it seems likely that in the beginning of an infection an increased PMN recruitment induced by LTB_4_ has a higher priority in the* Leishmania *survival strategy than the downregulation of PMN effector functions by LXA_4_.

Previously, our group identified a* Leishmania* promastigote-derived lipid mediator termed* Leishmania* chemotactic factor (LCF) [36]. On the one hand, comparable to LTB_4_, this lipid selectively recruits PMN and induces PMN-derived IL-8 which forms an amplifying loop for neutrophil recruitment [36, 38]. On the other hand, LCF shows analogies to resolution-phase LXA_4_ by simultaneously deactivating PMN and increasing uptake and intracellular survival of* Leishmania* as well as mediating its effects via the lipoxin A4 receptor (ALX/FPRL-1) [37]. Summing up, LCF shares features of both LTB_4_ and LXA_4_ and illustrates the strong impact of* Leishmania* on the lipid mediator environment. In line with our present results, LCF completes the view that* L. major* actively influences the early inflammation phase by changing towards a proinflammatory lipid mediator milieu for sufficient PMN recruitment while simultaneously dampening their activation in a LXA_4_-like fashion. Consequently our* in vitro* findings support the view that the induction of neutrophil-derived LTB_4_ is a part of the parasites survival strategy which enables the initial establishment of* Leishmania major* infection.

Experiments with* Anaplasma phagocytophilum* confirmed the important role of lipid mediators in the early phase of infection with intracellular pathogens. Comparable to* Leishmania major,* the observed increase of PMN-derived LTB_4_ and decrease of LXA_4_ production could enable the recruitment of large numbers of host cells and are likely to contribute to the establishment of* A. phagocytophilum* infection.

Since early after infection the survival of both pathogens depends on the recruitment of sufficient number of host neutrophils, the enhanced induction of LTB_4_ production appears to be crucial to successfully establishing the infection of the host. Moreover, the enhancing effect of LTB_4_ on the neutrophil phagocytic capacity [39] likely contributes to the entry of the pathogens into neutrophils. However, LTB_4_ also leads to enhanced antimicrobial effector functions such as the production of reactive effect oxygen species and phagosome-lysosome fusion [40, 41]. Since these functions can be detrimental for both* L. major* and* A. phagocytophilum, *the pathogens must possess effective mechanisms to evade these antimicrobial effector functions. Indeed, both* L. major* and* A. phagocytophilum *can effectively inhibit both the production of ROS [42, 43] and acidification of the phagolysosomes [30, 44].

For their transmission both* Leishmania* and* Anaplasma* are dependent on vectors. Salivary gland extracts (SGE) of* Lutzomyia longipalpis* sandflies that are insect vectors for* Leishmania *parasites were found to inhibit LTB_4_ production and LTB_4_-mediated chemotaxis [45]. Furthermore, SGE induces PGE_2_ which in turn can promote LXA_4_ synthesis. Consequently, SGE was shown to facilitate survival of* L. infantum *[46]. Similar to sandflies,* Ixodes ricinus*, the tick vector of* A. phagocytophilum*, secretes a leukotriene binding protein termed Ir-LBP that works as a “scavenger” for LTB_4_. By this effect tick saliva can decrease the number and activation level of PMN located at the tick bite site* in vivo* [47]. In summary there seems to be opposite LTB_4_-signaling of vector and pathogen due to different intentions. Whereas sandflies and ticks aim to secure blood meals by dampening inflammation, the pathogens need inflammation for sufficient host cell influx.

Other intracellular pathogens that are not dependent on neutrophils as host cells show different approaches to influencing the balance of lipid mediators. For example,* Mycobacterium tuberculosis* inhibits proinflammatory PGE_2_ and enhances lipoxin synthesis* in vivo* [48].* Toxoplasma gondii* has been shown to express its own 15-LOX leading to an increased lipoxin synthesis [49]. By induction of anti-inflammatory lipid mediators these pathogens seem to differ from the group of intracellular pathogens that use neutrophils as host cells. Nevertheless, these findings in concert with our data illustrate that manipulation of local lipid mediators by pathogens is a widespread phenomenon.

## 5. Conclusions

Taken together, our data support the view [50] that the neutrophil lipidome is a common target of intracellular parasites to modulate the influx and function of leukocytes at the site of infection. We showed that the vector-transmitted intracellular pathogens* Leishmania major* and* Anaplasma phagocytophilum* promote neutrophil-driven amplification of acute inflammation. This modulatory function likely contributes to the recruitment of host cells that are essential for the survival and multiplication of the pathogens. Further studies and* in vivo *models should confirm the impact of intracellular pathogens on host neutrophil lipidome. This could in turn provide a basis for therapeutic approaches to counteract the pathogens survival strategies at this level.
